# Expression of PD1/PDL1 in gastric cancer at different microsatellite status and its correlation with infiltrating immune cells in the tumor microenvironment

**DOI:** 10.7150/jca.40500

**Published:** 2021-01-18

**Authors:** Yan-li Wang, Yuehua Gong, Zhi Lv, Liang Li, Yuan Yuan

**Affiliations:** 1 Tumor Etiology and Screening Department of Cancer Institute and General Surgery, the First Hospital of China Medical University, Shenyang 110001, China.; 2 Key Laboratory of Cancer Etiology and Prevention in Liaoning Education Department, the First Hospital of China Medical University, Shenyang 110001, China.; 3 Key Laboratory of GI Cancer Etiology and Prevention in Liaoning Province, the First Hospital of China Medical University, Shenyang 110001, China.; 4 Department of Medical Oncology, the First Affiliated Hospital of Jinzhou Medical University, Jinzhou 121001, China.

**Keywords:** gastric cancer, tumor immune microenvironment, PD1, PDL1, CD8, CD68

## Abstract

**Objective:** The microsatellite status and tumor immune microenvironment have a remarkable influence on tumor immunotherapy. This study was performed to investigate programmed cell death protein 1/programmed death ligand 1 (PD1/PDL1) expression and their correlations with CD8+ T cell/CD68+ macrophage (CD68+ M) densities in gastric cancer (GC) at different microsatellite statuses.

**Methods:** The expression of MLH1, PMS2, MSH2, and MSH6 was detected via immunohistochemistry (IHC) to determine the microsatellite status in 215 GC samples obtained from surgical resections. Furthermore, the expression of PD1, PDL1, CD8, and CD68 was detected in the samples via IHC, and the differences and correlations in GC at different microsatellite statuses were then analyzed. PDL1 expression in tumor cells was labeled as PDL1[T], while expression of PD1 and PDL1 in tumor-infiltrating immune cells was labeled as PD1 and PDL1, respectively. Kaplan-Meier analysis was used to evaluate the significance of PD1/PDL1 expression in determining overall survival. Multivariate Cox regression analysis was performed using SPSS software. P-values were determined using the log-rank test.

**Results:** Our results indicated that PD1, PDL1[T], and PDL1 positivity rates were 59%, 35%, and 57% in 46 microsatellite unstable (MSI) GCs and 45%, 22%, and 40% in 169 microsatellite stable (MSS) GCs, respectively. Compared with MSS GC, PD1, PDL1[T], and PDL1 expression was higher in MSI GC (P = 0.109, 0.090, and 0.044, respectively). Additionally, CD8+ T cell and CD68+ M densities were higher in MSI GC than in MSS GC (P = 0.537 and <0.001, respectively). Additionally, CD8+ T cell/CD68+ M densities were evaluated according to tumor center and invasion front. We found that PD1 expression was significantly correlated with CD8+ T cell density at the invasion front of the MSI GC (P = 0.031), whereas PDL1 expression was significantly correlated to high CD68+ M density in the tumor center and invasion front of MSS GC (P = 0.001 and 0.014, respectively). Survival analysis showed that patients with PD1-positive and PDL1[T]/PDL1-negative GC had better prognosis (P = 0.012, 0.005, and 0.022, respectively). Multivariate Cox survival analysis showed that PDL1[T] was an independent prognostic factor for GC.

**Conclusion:** The results suggested that PD1/PDL1 expression and immune response varied at different microsatellite statuses in GC. PD1/PDL1 expression was correlated with CD8+ T cell/CD68+ M densities in GC at different microsatellite statuses, especially at the invasion front. The patients exhibiting high PD1/PDL1 expression or high CD8+ T cell/CD68+ M densities MSI GC might be potential beneficiaries of PD1/PDL1 immunotherapy.

## Introduction

Gastric cancer (GC) is the fifth most frequently diagnosed malignancy and the third leading cause of cancer-related death worldwide 1. Four molecular classifications of GC were proposed according to the comprehensive molecular characterization by TCGA, including Epstein-Barr virus-positive GC, microsatellite unstable GC (MSI), genomically stable GC, and chromosomal instability 2. As one of the four molecular subtypes, MSI GC accounts for 21.7% of all GCs and is caused due to the misfunction of the DNA mismatch repair (MMR) system. Compared with other molecular types, MSI GC has special characteristics, such as formation mechanisms, molecular compositions, immune microenvironments, and clinicopathological phenotypes. Knowledge of the unique biological characteristics of MSI GC is critical for its precise diagnosis and treatment.

Mounting evidence has shown that the tumor microenvironment plays an essential role in the progression of many solid tumors; tumor microenvironment is mainly orchestrated by tumor-infiltrating immune cells, including neutrophils, dendritic cells, macrophages, eosinophils, mast cells, and lymphocytes. The tumor microenvironment is indispensable for tumor progression, fostering proliferation, survival, and migration 3. Tumor-infiltrating lymphocytes (TILs, mainly CD8+ T lymphocytes) constitute the most essential effector cells of antitumor immunity 4. Tumor-associated macrophages (TAMs, which can be labeled by CD68 as CD68+ M) are also a significant component of tumor-infiltrating immune cells 5. Tumor-infiltrating immune cells and tumor cells interact with each other to maintain the balance of the tumor microenvironment. Therefore, knowledge of the heterogeneity of the tumor microenvironment, especially the immunocyte phenotypes related to therapeutic targets, can provide clues for new treatments for GC.

As one of the most promising immunotherapy approaches, programmed cell death protein 1 (PD1) and its ligand, programmed death ligand 1 (PDL1), have been used in the treatment of a variety of tumors in recent years 6-8. PD1, a member of the CD28 superfamily, is an important immunosuppressive molecule, mainly expressed in T, NK, and B cells surrounding tumor tissues 9. PDL1 is a 40 kDa type I transmembrane protein that is extensively expressed in immune, tumor, epithelial, and endothelial cells. In the tumor microenvironment, PDL1 is expressed in tumor cells interacting with PD1 expressed in T cells and inhibits the function of T cells. PD1/PDL1 immunotherapy can prevent the recognition between PD1 and PDL1, thus restoring the normal function of T cells 10, 11. Based on previous publications, the response rate to PD1/PDL1 immunotherapy appears to be associated with PDL1 expression levels 12, 13. Currently, the detection of PD1/PDL1 protein expression via immunohistochemistry (IHC) is routinely performed to select patients who will benefit from PD1/PDL1 immunotherapy 14. It has been found that 36% to 100% of patients with PDL1-positive tumor cells, detected via IHC, respond to PD1/PDL1 therapy; however, 0% to 17% of PDL1-negative patients also respond 15, suggesting that additional factors may be involved in predicting the response. Patients who could benefit the most from PD1/PDL1 immunotherapy required laminated anchoring. Therefore, additional biomarkers need to be further investigated, especially in the tumor immune microenvironment.

So far, the relationship among MSI characteristics of tumors, heterogeneity of the tumor microenvironment, and PD1/PDL1 immunotherapy has attracted increasing attention in clinical studies. A basic consensus has been reached to identify MSI status and PD1/PDL1 expression to select patients who would benefit from PD1/PDL1 immunotherapy 8, 14, 16, 17. However, PD1/PDL1 expression and its correlation with immune cell phenotypes in MSI GC remains unclear, as does the cells targeted by PD1/PDL1 immunotherapy. This study evaluated PD1/PDL1 expression in the microenvironment of MSI GC and analyzed its correlation with immune cell phenotypes to identify the cell types related to PD1/PDL1 expression in the microenvironment of MSI GC to better understand the interactions between tumor and immune cells and help select suitable target populations for future PD1/PDL1 immunotherapy in GC with different microsatellite statuses.

## Materials and Methods

### 1. Patients and clinical data collection

The samples and data of 215 patients who underwent surgical resection of primary GC at the first hospital of China Medical University and Jinzhou Medical University of Liaoning province between January 2012 and December 2018 were collected retrospectively. None of the patients had undergone neoadjuvant therapy. The histological diagnosis was confirmed by two pathologists independently, according to the World Health Organization criteria. Pathological Pathological Tumor Node Metastasis (TNM) staging was classified based on the Union for International Cancer Control (UICC; 8th edition, 2017). The study was approved by the Ethics Committee of the First Affiliated Hospital of China Medical University and Jinzhou Medical University. All participants enrolled in the study signed written informed consent forms.

### 2 Immunohistochemical staining and evaluation

#### 2.1 Immunohistochemical staining

The total number of cases in this study was 215. For each case, 9 consecutive sections were made for HE (Hematoxylin - Eosin) staining and immunohistochemical staining of 8 indexes including MLH1, MSH2, PMS2, MSH6, CD8, CD68, PDL1 and PD1. Because a few cases tissue is too little to the last 2-3 tissue section to complete the immunohistochemical evaluation. So some individual index staining is less than the total number of all cases. MLH1, MSH2, MSH6 and PMS2 evaluations were completed in all 215 cases, and 205 cases were evaluable for PD1, 202 cases for PDL1 [T]/PDL1, 205 cases for CD8, and 202 cases for CD68.

The primary antibodies used in this study (including anti-MLH1, -PMS2, -MSH2, -MSH6, -CD8, and -CD68 antibodies), DAB chromogenic solution, and UltraSensitive TMSP detection kit were purchased from Maixin (Maixin Inc., Fujian, China). Anti-PDL1 antibody 28-8 (ab205921), anti-PD1 antibody [EPR4877(2)] (ab137132), and Universal HIER antigen retrieval reagent (10X) (ab208572) were purchased from Abcam (Cambridge, UK).

IHC was performed on 4 μm-thick tissue sections mainly as previously described 18 at room temperature (18-30 °C) according to manufacturer's instructions. The primary antibodies (anti-MLH1, -PMS2, -MSH2, -MSH6, -CD8, and -CD68 antibodies) were ready for use. Anti-PD1 and anti-PDL1 antibodies were diluted at 1:260 and 1:600, respectively. Phosphate buffer solution (PBS) was used as the negative control, and positive staining of normal gastric interstitial immune cells was the internal control (MLH1, PMS2, MSH2, and MSH6).

#### 2.2 Immunohistochemical evaluation

Each section was first observed under a low-power microscope for the entire area. Positive staining areas were selected and observed under a high-power microscope. The evaluation was independently conducted by two senior pathologists.

##### 2.2.1 Microsatellite status assessment

MLH1, PMS2, MSH2, and MSH6 were all located in the GC cell nucleus. The tumor cell nuclei in brown yellow or brown granules were determined to be positive staining. Absence was determined when the tumor cell nucleus failed to stain. Microsatellite stability was determined when four proteins were positive, whereas MSI was determined when one or more proteins were absent. The microsatellite status was determined consistently by evaluating the expression of MLH1, PMS2, MSH2, and MSH6 in GC and adjacent peritumoral tissues simultaneously, although there were significant differences in the expression levels between them.

##### 2.2.2 PD1 and PDL1 expression evaluation

PD1 and PDL1 were located in the membrane and/or cytoplasm. PDL1 expression in tumor cells was labeled as PDL1[T], and expression of PD1 and PDL1 in tumor-infiltrating immune cells was labeled as PD1 and PDL1, respectively. We randomly selected five high-power fields (HPF, ×400, 0.025 mm^2^) from each sample, and manually counted 100 tumor or immune cells in each HPF and then calculated the percentage of positively stained cells. The mean value was adopted as the positive expression rate of PD1, PDL1[T], and PDL1. Positive staining was defined as more than 1% stained cells, regardless of cytoplasmic or membrane staining 14, 19.

##### 2.2.3 CD8 and CD68 expression evaluation

We randomly selected five HPFs (×400, 0.025 mm^2^) in the tumor center and invasive front, respectively, for each case, then manually counted and calculated the average number of stained CD8+ T cells and CD68+ M according to the median number of stained cells (CD8, 72.3/HPF; CD68, 43.2/HPF). Patients were divided into two groups: high and low density.

### 3. Statistical analysis

All statistical analyses were performed using SPSS software, Chicago, IL, USA (version 23.0). Intergroup comparison (associations between PD1/PDL1-positive expression, CD8/CD68-positive cell density, and MSI/MSS) was conducted using the χ2 test or Fisher's exact test. Kaplan-Meier analysis was used to evaluate the significance of different factors in determining overall survival. Multivariate Cox regression analysis was performed using SPSS software. P-values were determined using the log-rank test. P <0.05 was considered statistically significant.

## Results

### 1. Expression of MLH1, PMS2, MSH2, and MSH6 in GC and microsatellite status judgment

**Figure 1:**
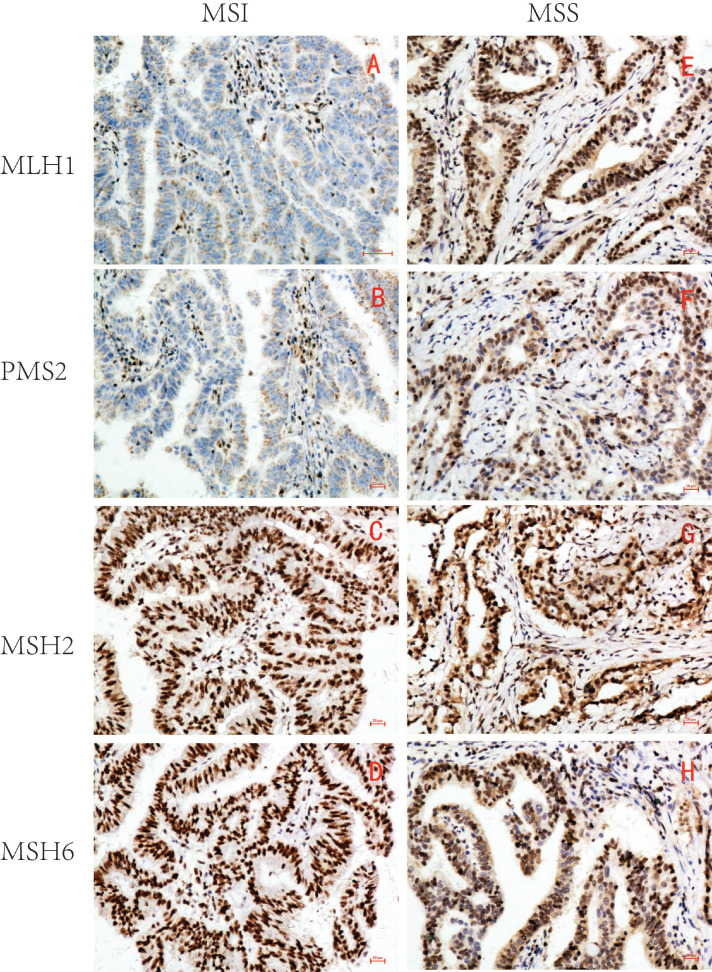
Representative immunohistochemical images of MSI/MSS GC (×200). MLH1, PMS2, MSH2 and MSH6 were all localized in GC cell nucleus. The brown yellow was considered positive, while the absence of staining was considered negative. MSI GC: MLH1 and PMS2 were negative(A,B), MSH2 and MSH6 were positive(C,D); MSS GC: MLH1, PMS2, MSH2 and MSH6 were all positive(E-H).

Based on the expression of MLH1, PMS2, MSH2, and MSH6, 215 samples were divided into two groups: 46 MSI (21.4%) and 169 microsatellite stable (MSS) (78.6%) (Table 1 and Figure 1).

### 2. PD1/PDL1 expression in MSI and MSS GC

**Figure 2:**
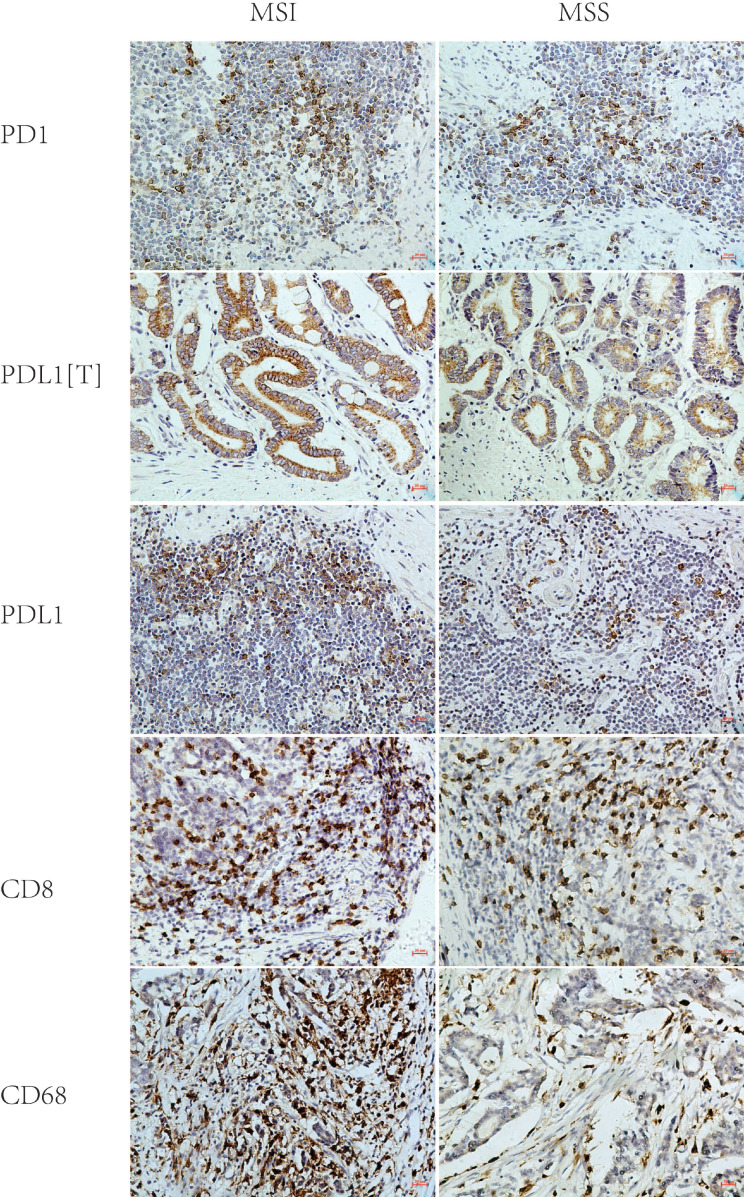
Representative immunohistochemical images of PD1, PDL1[T], PDL1, CD8 and CD68 in MSI and MSS GC (×200). PD1, PDL1[T], PDL1, CD8 and CD68 were all localized in the membrane and/or cytoplasm. PD1, CD8 and CD68 was all stained in tumor infiltrating immune cells, while PDL1 was stained in tumor cells (labeled as PDL1[T]) and tumor infiltrating immune cells (labeled as PDL1). PD1, PDL1[T] and PDL1 expression in MSI GC was greater than those in MSS GC (P=0.109,0.090 and 0.044, respectively). CD8+T cells/CD68+M density in MSI GC was higher than those in MSS GC (P=0.537 and <0.001, respectively).

PD1, PDL1[T], and PDL1 expression rates were 59%, 35%, and 57% in 46 MSI GC and 45%, 22%, and 40% in 169 MSS GC, respectively. Statistical analysis showed that the PDL1 expression rate in MSI GC was significantly higher than in MSS GC (P = 0.044), while the PD1/PDL1[T] expression rate in MSI GC was higher than in MSS GC but without statistical significance (P = 0.109 and 0.090) (Table 2 and Figure 2).

### 3. CD8+ T cell/CD68+ M densities in MSI and MSS GC

According to the median number of stained cells (CD8, 72.3/HPF; CD68, 43.2/HPF), patients were divided into two groups: high and low density. CD8+ T cell density in MSI GC was higher than in MSS GC but without statistical significance (P = 0.537). CD68+ M density in MSI GC was significantly higher than that in MSS GC (P < 0.001) (Table 2 and Figure 2).

### 4. Correlations between PD1/PDL1 expression and CD8+ T cell/CD68+ M densities in MSI and MSS GC

PD1 expression rate in the high CD8+ T cell density group was greater than that in the low-density group in MSI GC but without statistical significance (P = 0.058). PDL1 expression rate in the high CD68+ M density group was greater than that in the low-density group in MSS GC (P = 0.013). However, PDL1[T] expression was not correlated with CD8+ T cell/CD68+ M densities in MSI or MSS GC (Table 3).

### 5. Correlations between PD1/PDL1 expression and CD8+ T cell/CD68+ M densities at different locations in MSI and MSS GC

**Figure 3:**
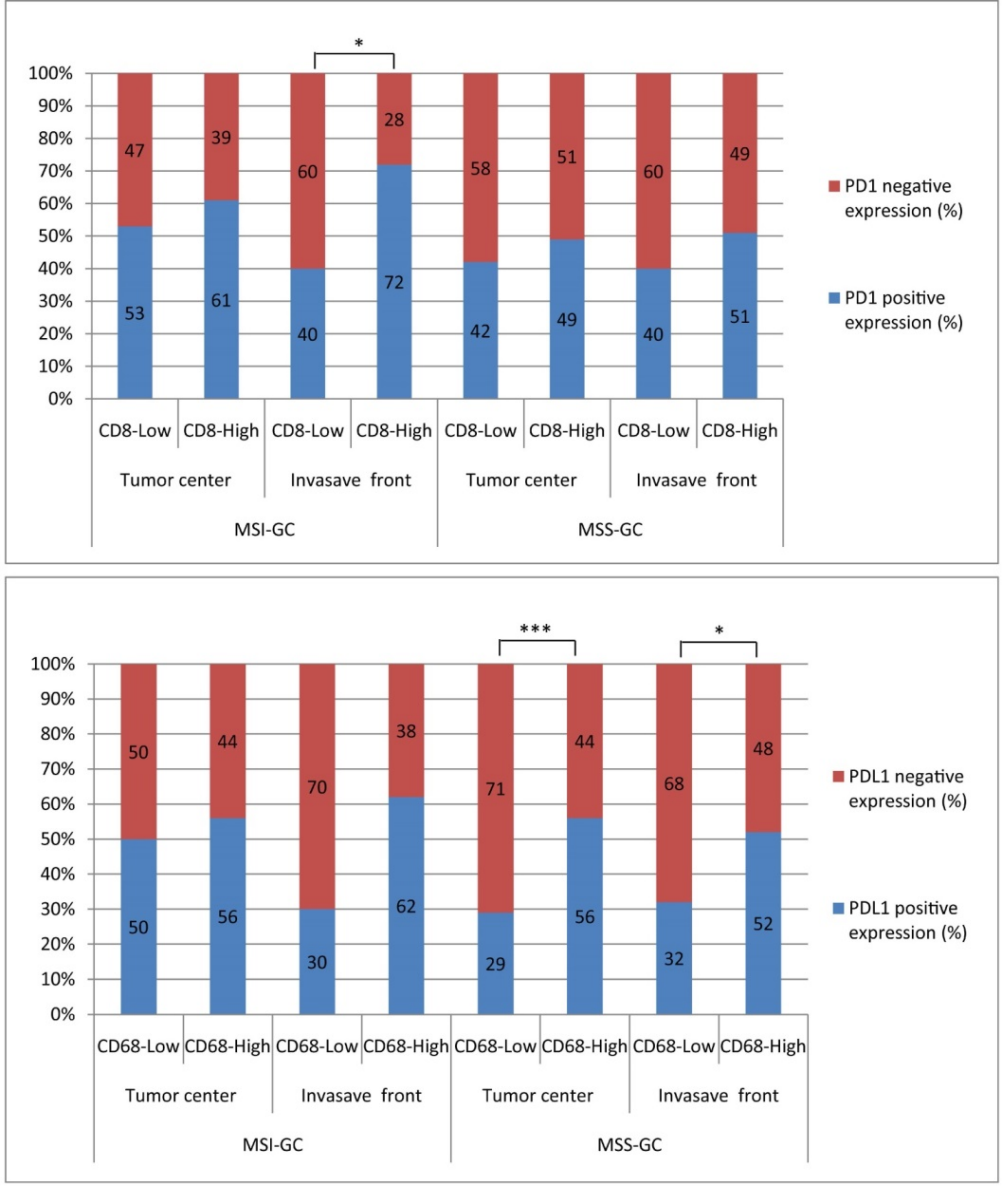
The correlations between PD1/PDL1 expression and CD8+T cells/CD68+M density at different locations in MSI and MSS GC. PD1 expression was significantly related to CD8+T cells high density at invasion front of MSI GC (P=0.031). PDL1 expression was significantly related to CD68+M high density in tumor center and invasion front of MSS GC (P=0.001 and 0.014, respectively).

PD1 expression was significantly correlated with CD8+ T cell density at the invasion front of MSI GC (P = 0.031). PDL1 expression was significantly correlated with high CD68+ M density in the tumor center and invasion front of MSS GC (P = 0.001 and 0.014, respectively). However, PDL1[T] expression was not correlated with CD8+ T cell/CD68+ M densities in the tumor center and invasion front of MSI or MSS GC (Table 4, 5 and Figure 3).

### 6. Correlations between PD1/PDL1 expression or CD8+ T cell/CD68+ M densities and prognosis in GC

Survival analysis showed that patients with MSI who were PD1 positive, PDL1[T] negative, and PDL1 negative had a better GC prognosis (P = 0.006, 0.012, 0.005, and 0.022, respectively). There were no correlations between CD8+ T cell/CD68+ M densities and prognosis in GC (P = 0.870 and 0.985, respectively). Multivariate survival analysis showed that microsatellite status, CD8+ T cell density, PDL1[T], TNM stage, infiltration depth, and vascular thrombi were all independent prognostic factors for GC.

## Discussion

In this study, we investigated PD1/PDL1 expression and its correlations with infiltrating immune cells in GC with different microsatellite statuses. It was found that both PD1/PDL1 expression and CD8+ T cell/CD68+ M densities were higher in MSI GC than in MSS GC. Furthermore, PD1/PDL1 expression was significantly correlated with high CD8+ T cell/CD68+ M densities, especially at the invasion front. These results may provide new information regarding the accurate stratification of target populations for PD1/PDL1 immunotherapy in GC.

A microsatellite is a simple, repetitive sequence evenly distributed in eukaryotic genomes under normal conditions. MSI refers to any change in the length of microsatellites resulting from insertion or deletion of repetitive units in a tumor and usually arises from germline mutations in the components of MMR genes (mainly MLH1, PMS2, MSH2, and MSH6) 20. Based on the expression of MLH1, PMS2, MSH2, and MSH6, 46 MSI GCs were detected in 215 GCs in this study; the proportion of MSI GC (21.40%) was similar to that in TCGA classification 2. We found that PD1/PDL1 expression in MSI GC was higher than that in MSS GC, suggesting that the immune response varied from microsatellite status in GC. Previous studies have reported that PD1/PDL1 immunotherapy shows little activity in unselected cancer 13, 21; however, significant clinical responses have been observed in patients characterized by MMR deficiency 16, 17. After radical surgery, patients with GC should be stratified and selected for postoperative adjuvant therapy. PD1 immunotherapy is a treatment option for chemotherapy-refractory GC, especially for PDL1-positive or MSI-High cancers 22, 23. PDL1 expression in tumor cells appears to be more common in MSI than in MSS GC 24, 25. Therefore, selecting beneficiaries of PD1/PDL1 immunotherapy according to microsatellite status in GC is a reasonable strategy.

The tumor microenvironment is primarily composed of TILs, especially CD8+ T cells and CD68+ TAMs. It has previously been reported that tumors with different microsatellite statuses may exhibit different immune microenvironments. MSI is associated with higher density TILs, possibly in response to the new environment created by neoantigens 26. Shin et al. found that the density of each subgroup of TILs varied from the microsatellite status of GC, and CD8+ T cell density was highly increased in MSI-H GC 27, 28. MSI-H GC was significantly correlated with CD8+ T cells 29. Furthermore, MSI tumors had more CD8+ T cell/CD68+ M in the epithelium, in contrast to MSS tumors 30. Intraepithelial CD68+ M density was significantly correlated with MSI 31. Additionally, we found that CD8+ T cell/CD68+ M densities were higher in MSI GC than in MSS GC, suggesting that the immune microenvironment was significantly different between MSI and MSS GC.

In our study, we analyzed the correlations between PD1/PDL1 expression and CD8+ T cell/CD68+ M densities in MSI or MSS GC. We found that PD1 expression was correlated with CD8+ T cell density in MSI GC, and PDL1 expression was significantly correlated with high CD68+ M density in MSS GC. It has been reported that the most common tumor-infiltrating immune cells are CD8+ T cells and CD68+ M, accounting for 15% and 13% of all tumor cells, respectively. Among the four molecular subtypes of GC classified by TCGA, MSI GC shows high-density infiltration of T cells and macrophages, among which T cells account for 30%-50%, while macrophages account for 20%, and 70% of MSI tumors express PDL1 32. PDL1 expression is strongly associated with a high density of TILs and MMR deficiency in GC 33. In a previous study, PDL1 expressed in cancer cells could inhibit the activation of CD8+ CTLs, allowing cancer cells to evade the immune monitor and survive 34. CXCL8 secreted by macrophages inhibits the function of CD8+ T cells and participates in the immunosuppression tumor microenvironment by inducing PDL1+ macrophages in GC 35. Furthermore, patients with GC exhibiting higher T cell infiltration showed increased PD1/PDL1 expression, indicating that an adaptive immune resistance mechanism might exist 36. In addition, MSI cancer may attract a large number of TILs and exhibit an active immune microenvironment, which leads to the significant upregulation of multiple immune checkpoint proteins, including PD1 and PDL1 26. This may explain why MSI tumors respond well to PD1/PDL1 immunotherapy.

The heterogeneity of immune cell distribution within tumors can influence the therapeutic response 37. The type and quantity of immune cells in the tumor center or invasion front of the tumor can vary greatly; this may explain the ability of these tumors to evade destruction by the human immune system. This phenomenon has been confirmed in colon cancer. Llosa et al. found that large numbers of PDL1+ immune cells, such as macrophages and TILs, were present at the invasion front of MMR defects in colon cancer 38. Korehisa et al. confirmed that PDL1, CD68+ M, and CD8+ T cell levels at the invasion frontier region were significantly higher than those in the tumor center of MSI-H colon cancer, and PDL1 was expressed in tumor cells and CD68+/CD163+ (M2) M at the invasion front of MSI-H colon cancer 39. However, such studies have not yet been conducted in GC. In our study, CD8+ T cell/CD68+ M densities were evaluated according to the focal location of cancer tissues, including the tumor center and invasion front. The correlations between PD1/PDL1 expression and CD8+ T cell/CD68+ M densities in different locations were further analyzed. We found a significant correlation between PD1/PDL1 expression and high CD8+ T cell/CD68+ M density, especially at the invasion front in MSI or MSS GC. Both pathways may contribute to tumor invasion and immune escape in MSI or MSS GC and thus may affect the efficacy of PD1/PDL1 immunotherapy.

This study further analyzed the relationship between PD1/PDL1 expression and CD8+ T cell/CD68+ M densities and prognosis. Until now, PD1/PDL1 expression in relation to prognosis in GC remains elusive. A meta-analysis showed that PDL1 negativity was associated with better overall survival in GC 24. Low PD1, PDL1, and CD8 mRNA levels were more significantly associated with a poor prognosis 40. A study revealed that CD68+ TAMs had no significant effect on OS 41. In this study, we found that patients with MSI who were PD1 positive, PDL1[T] negative, and PDL1 negative had a better prognosis in GC. Multivariate survival analysis showed that CD8+ T cell density and PDL1[T] were independent prognostic factors for GC. Our results are consistent with those of a previous study, and the differences involved remain to be further studied.

## Conclusion

In summary, we evaluated PD1/PDL1 expression and its correlation with CD8+ T cell/CD68+ M densities in MSI or MSS GC. The results showed that PD1/PDL1 expression and CD8+ T cell/CD68+ M densities were higher in MSI GC than in MSS GC, suggesting that PD1/PDL1 expression and the immune response varied based on microsatellite status in GC. Moreover, we found a significant correlation between PD1/PDL1 expression and high CD8+ T cell/CD68+ M density, especially at the invasion front in MSI or MSS GC, suggesting that they may participate in immune escape and affect the efficacy of immunotherapy. Patients with MSI with high PD1/PDL1 expression or high CD8+ T cell/CD68+ M densities might be potential beneficiaries of PD1/PDL1 immunotherapy. This study may provide a better understanding of the immune microenvironment in GC with different microsatellite statuses and may provide clues for seeking potential therapeutic targets and prognostic biomarkers for the treatment of GC in clinical settings.

## Figures and Tables

**Figure 4:**
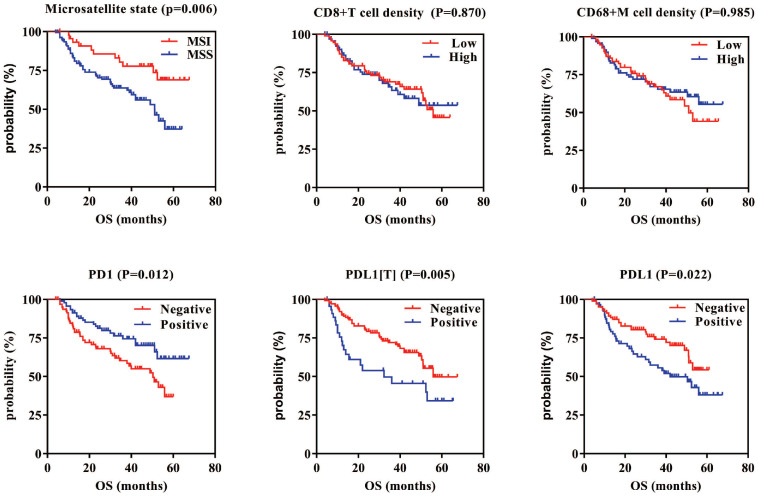
Kaplan-Meier overall survival curves of PD1/PDL1 expression or CD8+T cells/CD68+M density in GC. GC patients with MSI, PD1 positive, and PDL1[T]/PDL1 negative had better prognosis (P=0.006, 0.012, 0.005 and 0.022, respectively). There are no correlations between CD8+T cells/CD68+M density and prognosis in GC (P=0.870 and 0.985, respectively).

**Table 1:** Expression of MLH1, PMS2, MSH2 and MSH6 and microsatellite status in GC.

MLH1	PMS2	MSH2	MSH6	Samples (%)	MSI status	sum
+	+	+	+	169 (78.6)	MSS	169
-	+	+	+	6 (2.8)	MSI	46
+	-	+	+	22 (10.2)		
+	+	-	+	1 (0.5)		
+	+	+	-	7 (3.3)		
-	-	+	+	10 (4.6)		

GC, gastric cancer; MSI, microsatellite instability; MSS, microsatellite stability. MLH1, PMS2, MSH2 and MSH6 immunohistochemical evaluations were completed in all 215 cases.

**Table 2:** PD1/PDL1 expression and CD8+T cells/CD68+M density in MSI and MSS GC

	MSI (%)	MSS (%)	Sum	P
**PD1**				0.109
Negative	19(41)	87(55)	106	
Positive	27(59)	72(45)	99	
Sum	46(100)	159(100)	205	
**PDL1 [T]**				0.090
Negative	30(65)	121(78)	151	
Positive	16(35)	35(22)	51	
Sum	46(100)	156(100)	202	
**PDL1**				0.044
Negative	20(43)	94(60)	114	
Positive	26(57)	62(40)	88	
Sum	46(100)	156(100)	202	
**CD8**				0.537
Low	21(47)	83(52)	104	
High	24(53)	77(48)	101	
Sum	45(100)	160(100)	205	
**CD68**				<0.001
Low	7(16)	95(60)	102	
High	37(84)	63(40)	100	
Sum	44(100)	158(100)	202	

GC: gastric cancer; MSI: microsatellite unstable; MSS: microsatellite stable; CD68+ M: CD68+ macrophage; PD1: programmed cell death protein 1; PDL1, programmed death ligand 1; In all 215 cases of gastric cancer, 205 cases immunohistochemical sections were evaluable for PD1, 202 cases for PDL1 [T]/PDL1, 205 cases for CD8, and 202 cases for CD68.

**Table 3:** The correlations between PD1/PDL1 expression and CD8+T cells/CD68+M density in MSI and MSS GC

	MSI								MSS							
	CD8				CD68				CD8				CD68			
	Low (%)	High (%)	Sum	P	Low (%)	High (%)	Sum	P	Low (%)	High (%)	Sum	P	Low (%)	High (%)	Sum	P
**PD1**				0.058				0.443				0.086				0.405
Negative	12(57)	7(29)	19		4(57)	15(41)	19		50(61)	36(47)	86		54(57)	30(50)	84	
Positive	9(43)	17(71)	26		3(43)	22(59)	25		32(39)	40(53)	72		41(43)	30(50)	71	
Sum	21(100)	24(100)	45		7(100)	37(100)	44		82(100)	76(100)	158		95(100)	60(100)	155	
**PDL1[T]**				1.000				0.401				0.620				0.472
Negative	14(67)	16(67)	30		6(86)	24(65)	30		64(79)	56(76)	120		74(80)	44(75)	118	
Positive	7(33)	8(33)	15		1(14)	13(35)	14		17(21)	18(24)	35		19(20)	15(25)	34	
Sum	21(100)	24(100)	45		7(100)	37(100)	44		81(100)	74(100)	155		93(100)	59(100)	152	
**PDL1**				0.688				0.217				0.844				0.013
Negative	10(48)	10(42)	20		5(71)	15(41)	20		48(59)	45(61)	93		63(68)	28(48)	91	
Positive	11(52)	14(58)	25		2(29)	22(59)	24		33(41)	29(39)	62		30(32)	31(52)	61	
Sum	21(100)	24(100)	45		7(100)	37(100)	44		81(100)	74(100)	155		93(100)	59(100)	152	

GC: gastric cancer; MSI: microsatellite unstable; MSS: microsatellite stable; CD68+ M: CD68+ macrophage; PD1: programmed cell death protein 1; PDL1, programmed death ligand 1; In all 215 cases of gastric cancer, 203 cases immunohistochemical sections were evaluable for PD1 and CD8 simultaneously, 200 cases for PDL1 [T]/PDL1 and CD8 simultaneously, 199 cases for PD1 and CD68 simultaneously, and 196 cases for PDL1 [T]/PDL1 and CD68 simultaneously.

**Table 4:** The correlations between PD1/PDL1 expression and CD8+T cells density at different locations in MSI and MSS GC

	MSI-CD8								MSS-CD8							
	Tumor center (%)				Invasive front (%)				Tumor center (%)				Invasive front (%)			
	Low (%)	High (%)	Sum	P	Low (%)	High (%)	Sum	P	Low (%)	High (%)	Sum	P	Low (%)	High (%)	Sum	P
**PD1**				0.550				0.031				0.381				0.163
Negative	9(47)	10(39)	19		12(60)	7(28)	19		49(58)	37(51)	86		49(60)	37(49)	86	
Positive	10(53)	16(61)	26		8(40)	18(72)	26		36(42)	36(49)	72		33(40)	39(51)	72	
Sum	19(100)	26(100)	45		20(100)	25(100)	45		85(100)	73(100)	158		82(100)	76(100)	158	
**PDL1[T]**				0.831				0.832				0.691				0.427
Negative	13(68)	17(65)	30		13(65)	17(68)	30		64(76)	56(79)	120		64(80)	56(75)	120	
Positive	6(32)	9(35)	15		7(35)	8(32)	15		20(24)	15(21)	35		16(20)	19(25)	35	
Sum	19(100)	26(100)	45		20(100)	25(100)	45		84(100)	71(100)	155		80(100)	75(100)	155	
**PDL1**				0.345				0.947				0.148				0.743
Negative	10(53)	10(39)	20		9(45)	11(44)	20		46(55)	47(66)	93		49(61)	44(59)	93	
Positive	9(47)	16(61)	25		11(55)	14(56)	25		38(45)	24(34)	62		31(39)	31(41)	62	
Sum	19(100)	26(100)	45		20(100)	25(100)	45		84(100)	71(100)	155		80(100)	75(100)	155	

GC: gastric cancer; MSI: microsatellite unstable; MSS: microsatellite stable; CD68+ M: CD68+ macrophage; PD1: programmed cell death protein 1; PDL1, programmed death ligand 1; In all 215 cases of gastric cancer, 203 cases immunohistochemical sections were evaluable for PD1 and CD8 simultaneously in tumor center and invasive front, 200 cases for PDL1 [T]/PDL1 and CD8 simultaneously in tumor center and invasive front.

**Table 5:** The correlations between PD1/PDL1 expression and CD68+M cells density at different locations in MSI and MSS GC

	MSI-CD68								MSS-CD68							
	Tumor center (%)				Invasive front (%)				Tumor center (%)				Invasive front (%)			
	Low (%)	High (%)	Sum	P	Low (%)	High (%)	Sum	P	Low (%)	High (%)	Sum	P	Low (%)	High (%)	Sum	P
**PD1**				1.000				0.051				0.708				0.292
Negative	4(40)	15(44)	19		7(70)	12(35)	19		51(55)	33(52)	84		52(58)	32(49)	84	
Positive	6(60)	19(56)	25		3(30)	22(65)	25		41(45)	30(48)	71		38(42)	33(51)	71	
Sum	10(100)	34(100)	44		10(100)	34(100)	44		92(100)	63(100)	155		90(100)	65(100)	155	
**PDL1[T]**				0.361				0.361				0.102				0.290
Negative	8(80)	22(65)	30		8(80)	22(65)	30		74(82)	44(71)	118		71(81)	47(73)	118	
Positive	2(20)	12(35)	14		2(20)	12(35)	14		16(18)	18(29)	34		17(19)	17(27)	34	
Sum	10(100)	34(100)	44		10(100)	34(100)	44		90(100)	62(100)	152		88(100)	64(100)	152	
**PDL1**				0.743				0.147				0.001				0.014
Negative	5(50)	15(44)	20		7(70)	13(38)	20		64(71)	27(44)	91		60(68)	31(48)	91	
Positive	5(50)	19(56)	24		3(30)	21(62)	24		26(29)	35(56)	61		28(32)	33(52)	61	
Sum	10(100)	34(100)	44		10(100)	34(100)	44		90(100)	62(100)	152		88(100)	64(100)	152	

GC: gastric cancer; MSI: microsatellite unstable; MSS: microsatellite stable; CD68+ M: CD68+ macrophage; PD1: programmed cell death protein 1; PDL1, programmed death ligand 1; In all 215 cases of gastric cancer, 199 cases immunohistochemical sections were evaluable for PD1 and CD68 simultaneously in tumor center and invasive front, 196 cases for PDL1 [T]/PDL1 and CD68 simultaneously in tumor center and invasive front.

**Table 6:** Multivariate Cox regression analysis of the association of various clinicopathological features with overall survival in GC

Factors	Overall survival		
	RR	95%CI	P
Microsatellite status	3.053	1.782 - 5.231	<0.001
CD8+M cells density	1.795	1.214 - 2.655	0.003
CD68+M cells density	1.082	0.677 - 1.729	0.741
PD1 expression	1.205	0.802 - 1.809	0.369
PDL1[T] expression	2.696	1.579 - 4.602	<0.001
PDL1 expression	0.867	0.560 - 1.343	0.524
TNM stages	3.301	1.607 - 6.783	0.001
Infiltration depth	0.223	0.086 - 0.577	0.002
lymphatic metastasis	0.372	0.185 - 0.745	0.005
Vessel carcinoma embolus	0.711	0.462 - 1.094	0.121

RR: relative risk

## Abbreviations

GC: gastric cancer
MSI: microsatellite unstable
MSS: microsatellite stable
CD68+ M: CD68+ macrophage
PD1: programmed cell death protein 1
PDL1: programmed death ligand 1
